# Data-Driven Modeling Identifies TIRAP-Independent MyD88 Activation Complex and Myddosome Assembly Strategy in LPS/TLR4 Signaling

**DOI:** 10.3390/ijms21093061

**Published:** 2020-04-26

**Authors:** Xiang Li, Chuan-Qi Zhong, Zhiyong Yin, Hong Qi, Fei Xu, Qingzu He, Jianwei Shuai

**Affiliations:** 1 Department of Physics, Xiamen University, Xiamen 361005, China; 2 State Key Laboratory of Cellular Stress Biology, Innovation Center for Cell Signaling Network, School of Life Sciences, Xiamen University, Xiamen 361102, China; 3 Complex Systems Research Center, Shanxi University, Taiyuan 030006, China

**Keywords:** higher-order assembly strategy, LPS signaling, mathematical modeling, SWATH-MS, systems biology, TLR4 complexes

## Abstract

TLR4 complexes are essential for the initiation of the LPS-induced innate immune response. The Myddosome, which mainly contains TLR4, TIRAP, MyD88, IRAK1/4 and TRAF6 proteins, is regarded as a major complex of TLR4. Although the Myddosome has been well studied, a quantitative description of the Myddosome assembly dynamics is still lacking. Furthermore, whether some unknown TLR4 complexes exist remains unclear. In this study, we constructed a SWATH-MS data-based mathematical model that describes the component assembly dynamics of TLR4 complexes. In addition to Myddosome, we suggest that a TIRAP-independent MyD88 activation complex is formed upon LPS stimulation, in which TRAF6 is not included. Furthermore, quantitative analysis reveals that the distribution of components in TIRAP-dependent and -independent MyD88 activation complexes are LPS stimulation-dependent. The two complexes compete for recruiting IRAK1/4 proteins. MyD88 forms higher-order assembly in the Myddosome and we show that the strategy to form higher-order assembly is also LPS stimulation-dependent. MyD88 forms a long chain upon weak stimulation, but forms a short chain upon strong stimulation. Higher-order assembly of MyD88 is directly determined by the level of TIRAP in the Myddosome, providing a formation mechanism for efficient signaling transduction. Taken together, our study provides an enhanced understanding of component assembly dynamics and strategies in TLR4 complexes.

## 1. Introduction

Lipopolysaccharide (LPS) is a well studied component of the outer membrane of Gram-negative bacteria that can induce the systemic innate immune response [1]. LPS signaling initiation depends on the membrane-spanning complex formed by Toll-like receptor 4 (TLR4). TLR4 is one of the best-studied Toll-like receptors (TLRs) that recognize specific pathogen-associated molecular patterns (PAMP) [2]. As a critical component of immune responses, TLR4 dysregulation will promote aberrant cytokine production [3]. Upon LPS stimulation, TLR4 recruits its downstream signals through interactions with the Toll-interleukin-1 receptor (TIR) domains [4]. Myeloid differentiation primary response gene 88 (MyD88) is the first characterized downstream component of all the TLRs [5]. However, TLR4 is the only one that activates both MyD88 and TIR-domain-containing adapter-inducing IFN-β (TRIF)-dependent signaling pathways [6]. At present, the TIR domain-containing adaptor protein (TIRAP) is believed to act as a bridging adapter between TLR4 and MyD88 [7,8,9]. However, TIRAP is not essential for the activation of TLR5/7/9-driven inflammatory cytokine production. The recruited MyD88 mediates a signaling cascade including IL-1 receptor-associated kinase-1/4 (IRAK1/4) and TNF receptor-associated factor 6 (TRAF6), which ultimately leads to the activation of transcription factors such as nuclear factor-κB (NF-κB) and activator protein 1 (AP-1) to induce the production of pro-inflammatory cytokines such as TNF-α and IL-6. Although the LPS/TLR4 signaling pathway has been intensively studied [10], a quantitative description of how the components are associated and disassociated in TLR4 complexes to induce signaling transduction is still lacking.

Components in the TLR4 complexes of the LPS signaling are central mediators in determining the induction of the innate immune response [11]. The conventional understanding of the components in the complex are present at an equal ratio. However, recent studies revealed that the TIR domain of TIRAP can assemble with the TLR4 TIR-domain into filaments, and that TIRAP can induce the formation of large MyD88 TIR-domain assemblies [12]. It was also reported that MyD88 forms an oligomeric assembly with IRAKs through death domain (DD) interactions [13]. The crystal structure study shows that the MyD88-IRAK4 complex preferentially consists of 6–8 molecules of MyD88 and 4 molecules of IRAK4. Using the sequential window acquisition of the theoretical fragment ion spectra mass spectrometry (SWATH-MS) technique, the stoichiometry of the LPS-induced signaling complexes were recently studied [14], suggesting a higher-order assembly behavior of the components in TLR4 complexes as well. The complexes have a variable stoichiometry, and the higher-order structure of components in complexes plays important roles in signal processing and cell fate determination [15,16]. The question is thus, how higher-order component assemblies are formed in LPS/TLR4 signaling complexes to enable efficient downstream component activation under various circumstances.

Mathematical modeling of cellular signaling pathways is a powerful approach for quantitatively dissecting the underlying mechanisms [17,18]. Several models have been proposed to address the ratio between procaspase-8 and c-FLIP isoforms at the death-inducing signaling complex (DISC) in determining cell fate [19,20,21]. In particular, using a model-guided approach, it has been elucidated that the higher-order assembly of procaspase-8 in DISC is an essential step for the induction of apoptosis [22,23]. Furthermore, the linear and nonlinear regulatory responses controlled by the complexes with higher-order assembly have also been theoretically studied in synthetic networks [24]. However, to our knowledge, a quantitative investigation of LPS signaling that can give further insight into the complicated dynamics of complex assembly is still missing.

The SWATH-MS determined stoichiometry of TLR4 complexes in LPS signaling [14], which is currently the most sensitive data, provides a valuable resource for data-driven modeling [25]. To quantitatively study the aggregation processes of LPS-induced complexes, we constructed a SWATH-MS data-based model. The modeling result suggests that there is a TIRAP-independent MyD88 activation complex, in which TRAF6 is not included. We also showed how the components are distributed in TIRAP-dependent and -independent MyD88 complexes. The results suggest that the component distribution is LPS stimulation-dependent. Further analysis indicates that the higher-order assembly of components in the complex are stimulus-strength-dependent as well, implying a possible mechanism link between the component distribution and higher-order assembly behavior. Our study provides new mechanistic insights into the TLR4 complex components and their assembly strategy that enables efficient transduction for LPS signaling.

## 2. Results

### 2.1. SWATH-MS Data-Based Modeling of LPS-Induced Myddosome Assembly

**Figure 1:**
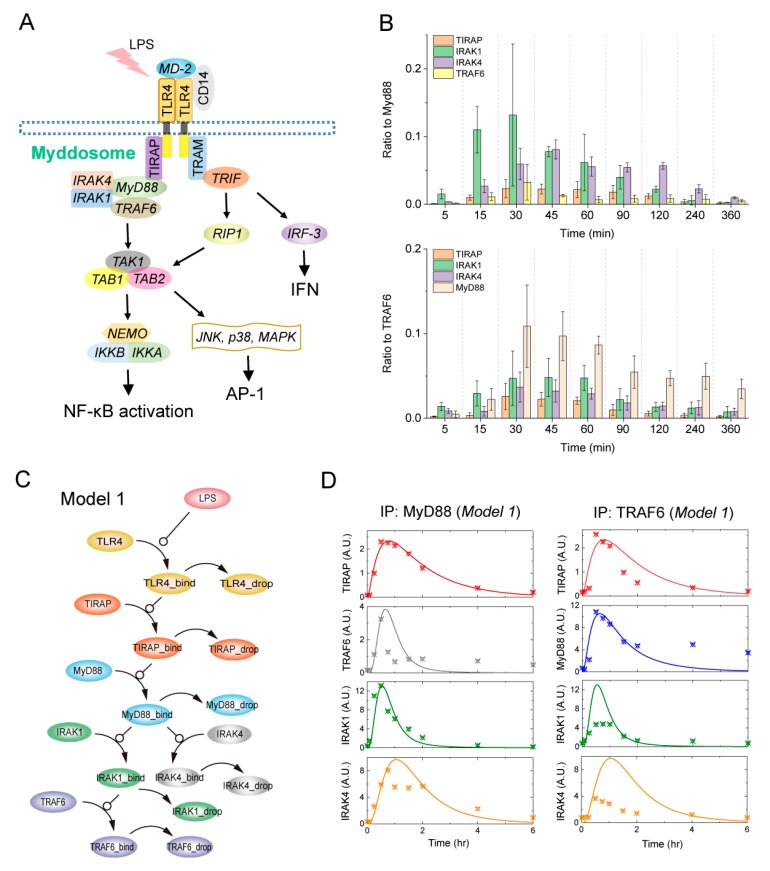
LPS-induced TLR4 complexes signaling transduction and model construction. (**A**) Overview of LPS/TLR4 signaling. (**B**) The stoichiometry of MyD88 and TRAF6 complexes determined by SWATH-MS. Data were obtained from ref [14]. (**C**) Kinetic mathematical model of the LPS-induced components association and disassociation in Myddosome (Model 1). (**D**) Simulation using Model 1 (lines) and SWATH-MS data (dots) of the time-course responses. Left and right panels respectively correspond to the major component dynamics in the MyD88 and TRAF6 complexes.

Upon LPS stimulation, interactions among LPS, MD-2 and CD14 occur, which facilitate the transfer of LPS to bind TLR4 (Figure 1A). Oligomerization of TLR4 is then initialized and the downstream adaptors are recruited to form complexes. The TIR domain of TLR4 is required for recruiting the TIR domain-containing adaptor proteins, i.e., MyD88, TIRAP, TRAM and TRIF [26]. Through the death domain, the recruited MyD88 can further bind to the death domain-containing kinases, IRAK1 and IRAK4. As the downstream of IRAK1, TRAF6 is also essential in the complex formation. TLR4, TIRAP, MyD88, IRAK1/4 and TRAF6 are the major constituents of the Myddosome.

The TLR4 complexes are divided into two signaling branches: The MyD88-dependent pathway and the MyD88-independent pathway. The MyD88-dependent pathway is activated through the signaling branch of the Myddosome, which is responsible for the TAK1-TAB1-TAB2 complex formation. The TAK1 complex then activates downstream IκB kinase (IKK) and mitogen-activated protein kinase (MAPK) pathways. IKK activation determines the translocation of NF-κB, which maintains the gene expressions of pro-inflammatory and other immune related cytokines. Another transcription factor, AP-1, which also mediates pro-inflammatory cytokine expression, is downstream of MAPK pathway activation. The MyD88-independent pathway is activated through the branch of TRAM-TRIF. Activated TRIF can recruit and mediate receptor-interacting protein 1 (RIP1) through the Rip homotypic interaction motif (RHIM). RIP1 is also an important mediator of the TAK1 complex, thereby regulating immune responses through activating NF-κB and MAPK pathways. TRIF can activate interferon regulatory factor 3 (IRF3), which is upstream of IFN signaling, leading to the induction of a number of IFN-inducible genes.

The endogenous MyD88 and TRAF6 complexes were purified and quantified from macrophage cell line RAW 264.7 in previous experiments [14]. The cells were treated with LPS for 10 different time periods and the major known components were detected and quantified (Figure 1B). LPS was derived from Gram-negative *Escherichia coli* and the stimulation concentration was 100 ng/mL. Changes in the stoichiometry of the MyD88 and TRAF6 complexes at the corresponding time periods (5, 15, 30, 45, 60, 90, 120, 240, 360 min) are respectively presented in the upper panel and down panel of Figure 1B. As the results show, SWATH-MS-based proteomics does not only allow for quantification of proteins across multiple time periods, but also provides protein stoichiometry in the MyD88 and TRAF6 complexes for one time period.

The accurate stoichiometry of proteins serves as a solid basis for experiment-based modeling to better understand the LPS-induced Myddosome assembly processes. We therefore developed a mathematical model (Model 1) that describes the current understanding of the Myddosome assembly processes (Figure 1C). The processes in Model 1 can be systematically represented by a set of association and disassociation biochemical reactions (Table 1). These reactions are subsequently formulated by a cast of ordinary differential equations (ODEs), which are represented by compounds and kinetic parameters based on the law of mass action (Table S1). The kinetic parameters were determined by a global optimization method that minimized the deviation between simulation results and MS data.

Simulation curves of the components in the complexes are compared with the MS data (Figure 1D). As shown in Figure 1D, Model 1 can generally reproduce the stoichiometry of major components in MyD88 (Figure 1D, left panel) and TRAF6 complexes (Figure 1D, right panel). The components in both MyD88 and TRAF6 complexes achieve the maximum amounts within 1 h and then dissociate slowly (Figure 1D). The results also show that the ratio among TIRAP, MyD88, IRAK4, IRAK1 and TRAF6 is about to 1:6:5:6:2 at the peak of Myddosome formation. The Myddosome completely disappears after about 6 h of LPS treatment, since the components can hardly be detected in complexes. However, the dynamics for some components in the complexes cannot be well-reproduced using Model 1, such as TRAF6 in the MyD88 complex, and MyD88 and IRAK1/4 in the TRAF6 complex. Therefore, we are missing some essential assembly processes in Model 1, which are different from the current understanding.

### 2.2. TIRAP-Independent MyD88 Activation Complex Formation upon LPS Stimulation

Studies have shown that the five components (TIRAP, MyD88, IRAK1, IRAK4 and TRAF6) are upstream-downstream relations and essential for signaling transduction in the Myddosome [26]. Thus, we developed Model 1 based on the consideration that all key components should be assembled into the Myddosome (Figure 1C). If the five components are indeed to be only assembled into the Myddosome upon LPS stimulation, the response dynamics of the components in both MyD88 and TRAF6 complexes should be the same. As shown in Figure 1D, simulation responses of the components (TIRAP, IRAK1 and IRAK4) in both complexes are the same. However, the MS data of IRAK1 and IRAK4 are an obvious discrepancy in the MyD88 complex and the TRAF6 complex. The amounts of IRAK1 and IRAK4 in the MyD88 complex are approximately 2–3 fold those in the TRAF6 complex at the peak of the Myddosome formation. Furthermore, the MS data of TRAF6 and MyD88 in the complexes suggests that even the Myddosome disappears later, the MyD88 and TRAF6 are still bound together.

**Figure 2:**
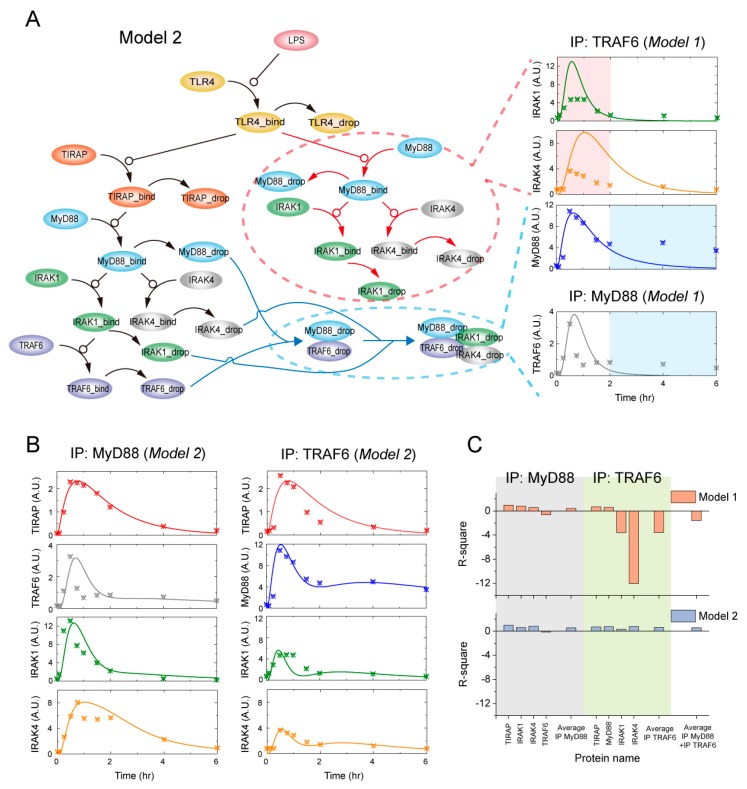
LPS induced the formation of the TIRAP-independent MyD88 activation complex. (**A**) Discrepancy between the Model 1 simulation results and SWATH-MS data suggest TIRAP-independent MyD88 complex and MyD88-TRAF6 complex formation upon LPS stimulation. (**B**) Simulation using Model 2 (lines) and SWATH-MS data (dots) of the time-course responses. (**C**) Comparison results of the R-square values between Model 1 (upper panel) and Model 2 (down panel).

The dynamic responses of TIRAP are the same in the MyD88 and TRAF6 complexes, while the downstream IRAK1 and IRAK4 display different responses (Figure 1D). The amounts of IRAK1 and IRAK4 in the MyD88 complex are larger than those in the TRAF6 complex, suggesting that a TIRAP-independent MyD88-IRAK1-IRAK4 complex should exist upon LPS stimulation. Furthermore, a MyD88-TRAF6 complex should also be formed when the Myddosome is disassembled. We therefore refined Model 1 by introducing the TIRAP-independent MyD88 activation complex and a MyD88-TRAF6 complex formation processes when the Myddosome is disassembled (Figure 2A). The additional TIRAP-independent MyD88 activation cascade might possibly explain the obvious discrepancy of IRAK1/4 in the TRAF6 complex between MS data and Model 1 simulation results (red dashed circle in Figure 2A), while the MyD88-TRAF6 complex subsequently dropped from the Myddosome (blue dashed circle in Figure 2A) could possibly explain the discrepancy of MyD88 and TRAF6 in the complexes.

We refined Model 1 by adding a set of biochemical reactions (Table 2) that describe the association/disassociation processes in the red and blue dashed circles of Figure 2A, which is named as Model 2. The kinetic parameters in Model 2 were re-determined by the global optimization method (Table 2) and the corresponding ODEs are presented in Table S2. As indicated in Figure 2B, Model 2 can well-reproduce the dynamic responses of all the key components in both MyD88 and TRAF6 complexes. In addition, we calculated the square of the correlation (R-square) between the MS data and the simulated results for the two models (Figure 2C). As expected, small R-square values were observed for the MyD88 complex components and their average for Model 1 and Model 2 (gray region in Figure 2C). However, relatively large negative R-square values were obtained for IRAK1/4 and the average of all the components in the TRAF6 complex in Model 1 (green region in Figure 2C). As a contrast, the R-square values were close to 1 for all the components and their average in the TRAF6 complex in Model 2 (green region in Figure 2C). Finally, the average R-square of all the components in the two complexes changed from a negative value in Model 1 to a positive value close to 1 in Model 2. Therefore, the comparison of the R-square values overwhelmingly support our conclusion that an additional TIRAP-independent MyD88 activation complex and the subsequently dropped MyD88-TRAF6 complex when the Myddosome is disassembled should be included in the LPS/TLR4 signaling pathway.

### 2.3. Distribution Strategy of Proteins in Complexes Determined by LPS Stimulation Strength

The above analysis reveals the TIRAP-dependent and -independent MyD88 activation cascades upon LPS stimulation. Studies have shown that the number of proteins in the signaling complexes is the key factor in cell fate determination [19,20,21]. We can therefore dissect the percentage of different component distributions in complexes using Model 2.

**Figure 3:**
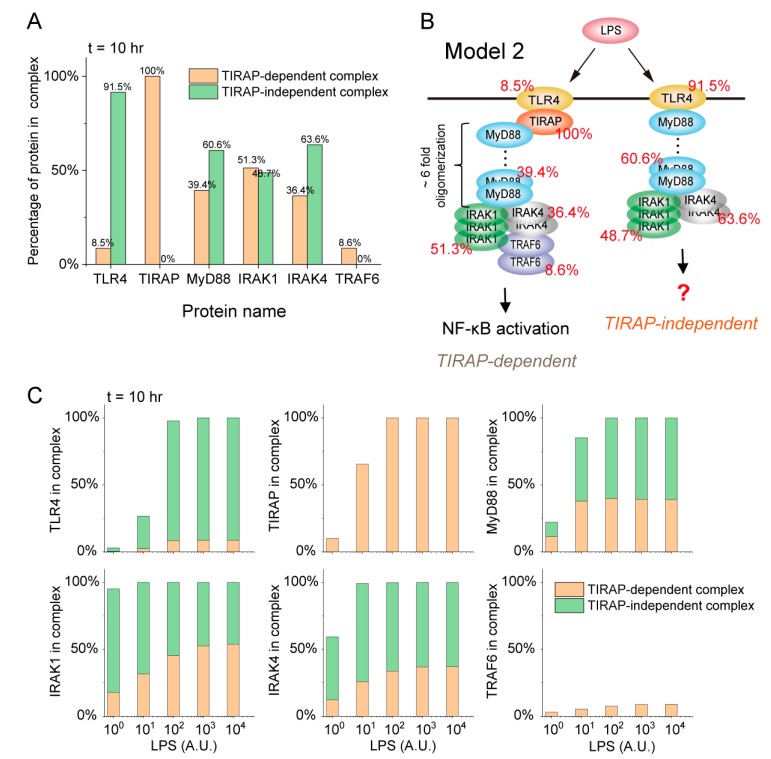
Components distribution strategy in the TLR4 complexes. (**A**) Percentage of components distribution in TIRAP-dependent and -independent MyD88 complexes. To calculate the total amount of each component recruited into the complexes, we did not consider the components disassociation processes and set the corresponding parameters to 0. The 10-hr LPS stimulation results were calculated as each component reached its maximum amount. (**B**) Schematic of the components distribution in complexes. (**C**) Percentage of components distributions in TIRAP-dependent and -independent MyD88 complexes upon different LPS stimulation-strengths.

The percentage of each protein recruited into the complexes can be calculated by P_COM_/P_INI_, where P_COM_ is the total amount of each protein recruited into each complex after LPS stimulation and P_INI_ represents the initial amount of each protein in the model. The 10-h LPS stimulation results were calculated as the dynamic behavior of components in a stable state where each component can reach its maximum amount. The percentage distribution of the six proteins that are respectively recruited into the TIRAP-dependent and -independent MyD88 complexes upon 10^2^ A.U. LPS stimulation are calculated and shown in Figure 3A. The results indicated that only a small amount of TLR4 (8.5% of the initial amount) is required for the Myddosome, i.e., TIRAP-dependent MyD88 complex assembly, while a large amount of TLR4 (91.5%) is recruited to form the TIRAP-independent MyD88 complex (Figure 3B). For MyD88, 39.4% of the initial amount is recruited into the Myddosome, and more MyD88 (60.6%) is recruited into the TIRAP-independent MyD88 complex. IRAK1 almost distributes averagely in TIRAP-dependent and -independent MyD88 complexes, while a large amount of IRAK4 (63.6%) is collected in the TIRAP-independent MyD88 complex. Hence, a quantitative picture of component distributions in the TIRAP-dependent and -independent MyD88 complexes upon LPS stimulation can be derived and the result is presented in Figure 3B. As the MS data shows (Figure 2B, right panel), the ratio of MyD88 to TIRAP is about 6:1 when the TRAF6 complex reaches its maximum, suggesting an approximate 6-fold oligomerization of MyD88 in the TRAF6 complex (Figure 3B). TRAF6 is an essential component in the Myddosome for the activation of NF-κB and MAPK. Then, the result raises a question of how the TIRAP-independent MyD88 complex executes biological function without TRAF6 but with a large amount of TLR4, MyD88 and IRAK4.

The responses of the biological system are mostly stimulus-strength-dependent. We next used Model 2 to study whether a distribution strategy of components in complexes upon different strengths of LPS stimulation exists (Figure 3C). The results suggest that all of the components in the Myddosome increase as the LPS stimulation strength increases. Different components in the Myddosome show different threshold responses to LPS stimulation strength. TLR4 and TIRAP reach their maximum amounts in the Myddosome upon 10^2^ A.U. LPS stimulation. However, the threshold of LPS stimulation strength is 10 A.U. for MyD88, while the threshold strength is 10^3^ A.U. for IRAK1/4 and TRAF6. As the result suggested, no matter how strong the LPS strength is, only a small amount of TLR4 (~10% of the initial amount) is required for Myddosome assembly. All TIRAP is recruited into the Myddosome to execute biological function upon strong LPS stimulation, but only a small amount of TRAF6 (~10%) is required.

The Myddosome and the TIRAP-independent MyD88 complex compete for recruiting the IRAK1/4. Upon weak LPS stimulation (1–10 A.U.), most IRAK1 (~75%) is recruited into the TIRAP-independent MyD88 complex and ~25% IRAK1 is located in the Myddosome. IRAK1 is equally distributed in the two complexes upon strong LPS stimulation (10^2^–10^4^ A.U.). Similarly, distributions of IRAK4 in the TIRAP-independent MyD88 complex and Myddosome are respectively decreased and increased when LPS stimulation strength increases. The amount of IRAK1/4 in complexes will directly determine the dynamics of downstream signaling. These results indicate that the undetermined biological function controlled by TIRAP-independent MyD88 complex could be impaired with increasing LPS stimulation strength, while the function mediated by the Myddosome could be enhanced. Thus, the optimal LPS stimulation for the two complexes to execute their biological functions are at a weak and strong strength, respectively. Indeed, earlier studies have shown that Myddosome-mediated NF-κB signaling has no response to a low dose (i.e., 10^−2^–1 ng/mL) of LPS stimulation in RAW 264.7 cells, but is completely activated upon a high-dose (i.e., 10^2^–10^4^ ng/mL) [27]. However, the undetermined function controlled by the TIRAP-dependent MyD88 complex at a weak LPS stimulation strength needs further research. Overall, the distribution strategy of IRAK1/4 in TLR4 complexes is strongly dependent on LPS stimulation strength. Thus, the commonly held view of LPS-induced TLR4 complexes determines the execution of downstream functions; thereby governing distinct cell fates, which should now be re-evaluated.

### 2.4. Higher-Order Assembly Strategy of MyD88 Determined by the TIRAP Level in the Myddosome

**Figure 4:**
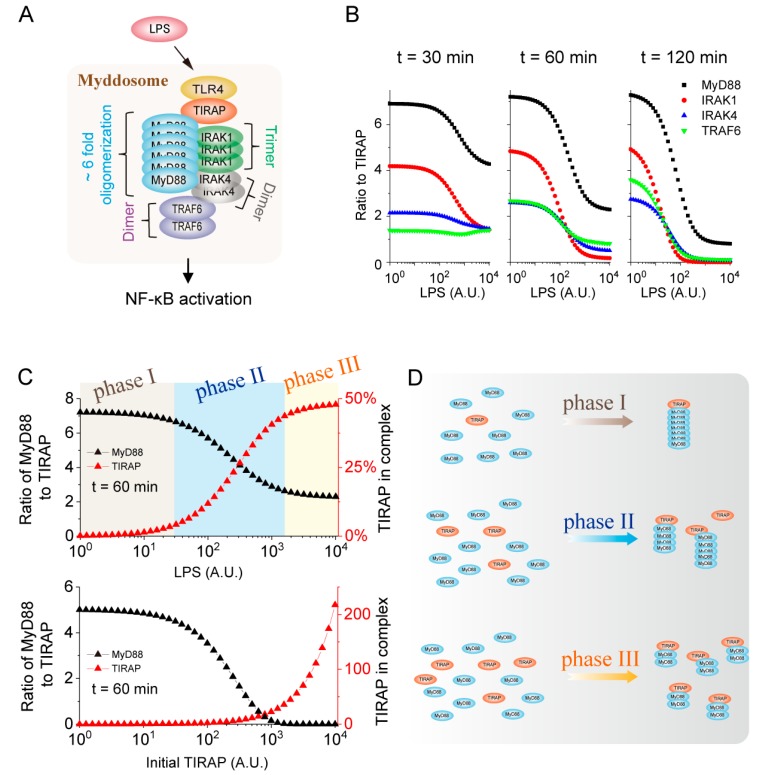
Higher-order assembly of the components in the Myddosome. (**A**) Schematic of the higher-order assembly of the components in the TLR4 complexes. There is about 6-fold more MyD88 than TIRAP in the Myddosome. (**B**) Ratio of the components to TIRAP in the Myddosome upon different LPS stimulation-strength at 30, 60 and 120 min, respectively. (**C**) The changes of the ratio of MyD88 to TIRAP and the amount of TIRAP in the Myddosome with LPS stimulation-strength (upper panel) and TIRAP initial level (down panel), respectively. (**D**) Diagram of the three phases that show that MyD88 higher-order behaviors are TIRAP amount-dependent.

Besides the distribution of the amount of the components, our MS-based model can also dissect the stoichiometry of components in complexes. As shown in Figure 3B, the ratio of TIRAP:MyD88 is about 1:6 in the TRAF6 complex upon 10^2^ A.U. LPS stimulation (Figure 4A). We thus attempted to explore whether and how the LPS stimulation strength affects the stoichiometry of components in the Myddosome.

The ratio of all the downstream components to TIRAP gradually decreases with the increasing strength of LPS stimulation (Figure 4B). These results indicate that the decreasing tendency of the ratio becomes more obvious with time. When the LPS level increases from 1 to 10^4^ A.U., the ratio of MyD88 decreases from about 7 to 4 at 30 min, while the ratio decreases from about 7 to 1 at 120 min. Since both the distribution (Figure 3C) and stoichiometry (Figure 4B) of components in the Myddosome are LPS stimulation-dependent, we therefore sought to investigate the possible mechanism between components distribution and stoichiometry.

MyD88 is directly recruited by TIRAP through the TIR domain, and invariably has a maximum ratio to TIRAP of all the components in the Myddosome upon different LPS stimulation strengths (Figure 4B). We therefore compared the change curves of the ratio of MyD88 to TIRAP and the amount of TIRAP in the Myddosome with LPS (Figure 4C, upper panel). The tendency of the two response curves are highly correlated and can be divided into three phases, i.e., phase I, II and III, which correspond to the LPS stimulation ranges at weak, middle and strong strength, respectively.

In phase I, the recruited TIRAP, which is essential for downstream MyD88 activation in the Myddosome, remains at a low level. Meanwhile, the ratio of MyD88 to TIRAP remains at a high level, suggesting that MyD88 forms a higher-order assembly state with 7-fold more MyD88 than TIRAP in the Myddosome (Figure 4D, upper panel). In phase II, the amount of TIRAP that activates MyD88 increases while the ratio of MyD88 decreases with the increasing LPS strength. The increased amount of TIRAP corresponds to a 2 to 4-fold decrease of the MyD88 ratio (Figure 4D, middle panel). In phase III, the amount of TIRAP reaches the threshold level while the ratio stays at a low level, indicating that the dimerization of MyD88 occurs with the highest level of TIRAP (Figure 4D, lower panel).

Since MyD88 recruitment is directly determined by TIRAP, we speculated that the higher-order aggregation form of MyD88 is determined by the level of TIRAP in the Myddosome. To validate this, we studied how the TIRAP amount in the Myddosome and the ratio of MyD88 to TIRAP change at different initial levels of TIRAP (Figure 4C, lower panel). As expected, the increase of TIRAP initial level leads to the increase of TIRAP in the Myddosome, which increases rapidly when the level is above 10^3^ A.U. of the initial TIRAP. The ratio of MyD88 to TIRAP decreases with the increase of the TIRAP initial level and is highly correlated with the amount of TIRAP in the Myddosome. The higher the TIRAP amount in Myddosome is, the less the higher-order aggregation behavior of MyD88 is.

Therefore, we conclude that the downstream higher-order structure of MyD88 is TIRAP level dependent in the Myddosome. The result provides new insight into the principle for MyD88 higher-order formation in the Myddosome. Different formation strategies of MyD88 in the Myddosome under different conditions might enable the LPS signaling for convenient and efficient transduction, which needs to be further studied with a more comprehensive model.

## 3. Discussion

Although the LPS signaling pathway has been extensively studied, the quantitative elucidation of the components and their assembly behaviors are poorly understood. In this study, we developed a SWATH-MS data-based mathematical model to provide insight into this question. The quantitative data of proteins obtained by SWATH-MS serve as a solid basis for experiment-based modeling using ODEs. Currently, most models have been constructed based on semi-quantitative experimental data (e.g., Western-blot), which is typically inaccurate and within a low dynamic range. With the scarcity and incomplete nature of data, model-based mechanistic investigation of cellular signaling regulation might be partially helpful or even sometimes misleading. In contrast, the SWATH-MS-based model of the signaling pathway can present precise biochemical mechanism descriptions and quantitative predictions. Actually, a large abundance of proteins in LPS signaling have been identified by the SWATH-MS [14]. Since the functions and interactions for many proteins are uncertain, we only considered the well-characterized key proteins in our study. The capability of the MS data-driven model need to be further modified with increasing knowledge for comprehensively understanding LPS signaling transduction.

TIRAP was originally thought to be involved in the MyD88-independent module of the TLR4 signaling [28,29]. However, similarly to the MyD88 knockout, TIRAP knockout macrophages delay TLR4-mediated NF-κB and JNK activation, and impair inflammatory cytokine production, which does not impair IFN-induced gene expression [7,8]. Thus, TIRAP was then determined as an essential component in the Myddosome for the MyD88-dependent module activation. In this study, we report that, for the first time to our knowledge, a TIRAP-independent MyD88 complex could be formed in the TLR4 signaling pathway upon LPS stimulation. Although there is no direct experimental evidence for validation, several presented studies indirectly support this conclusion. Structural study of TIR-domain-assembly formation reveals that TIRAP promotes MyD88 to assemble into large structures for signaling transduction [12]. However, the study also indicated that MyD88 clusters spontaneously at high expression levels without TRIAP upon TLR4 stimulation. Furthermore, another study also proved that MyD88 is absolutely required for the macrophage cytokine response, but the requirement of TIRAP could be overcome by mediating the concentrations of the components [30].

Our results indicate that TRAF6 is not included in the TIRAP-independent MyD88 complex, but TRAF6 is required for NF-κB and JNK activation in the Myddosome. Thus, unlike the Myddosome, the TIRAP-independent MyD88 complex may execute some other unknown biological functions. The former conclusion of TIRAP required for MyD88 activation is mostly obtained from detecting NF-κB and JNK activation [9], while herein the TIRAP-independent MyD88 complex might regulate other downstream activation cascades, such as caspase-8 mediated apoptosis [31]. The previous studies cannot deny the formation of the TIRAP-independent MyD88 complex without detecting its unknown functions. TIRAP knockout mice showed normal immune responses to many TLR (TLR3/5/7/9) ligands for the induction of inflammatory cytokine production [10]. MyD88 can directly bind to these TLRs through the TIR domain. As a member of the TLR family, there is no evidence showing that TLR4 cannot directly recruit MyD88 into a complex. Currently, the challenge is to experimentally validate the existence of the TIRAP-independent MyD88 complex and to determine the complex biological functions.

Several studies have shown that cell fate is determined by the stoichiometry in complexes. Both the experiment and model analysis proved that the cell survival/apoptosis decisions are determined by the balance of c-FLIPL and DISC [19,20,21]. In addition, the cell survival/necrosis outcomes proved to be controlled by the stoichiometry of RIP1 and RIP3 in the necrosome [32]. Our study provides a quantitative picture of the assembly dynamics of components in the TLR4 signaling complexes. With this model, we determined the distribution of components in different complexes and further showed that the distribution is LPS stimulation-dependent (Figure 3). Different distributions will induce a variable stoichiometry of the complexes, thereby showing various regulation strategies on the downstream signals, and ultimately resulting in distinct cell fate upon different stimulation strength. The effects of the variable stoichiometry in TLR4 complexes on downstream activation, such as NF-κB, JNK and IFN, can be further explored through developing a more comprehensive model in our future work.

Higher-order complexes are the oligomers in which a large number of monomers are contained in a complex. Recent studies advanced our understanding of their ability to execute threshold behavior, signal amplification and noise reduction functions of signal transduction [15,16]. Furthermore, phase separations in cells appear to be directly driven by the higher-order assembly of complexes [33]. Our study indicated that about 7 MyD88 molecules and 3 IRAK4 molecules bind to 1 TIRAP upon weak LPS stimulation (Figure 4B), which is supported by a TIR-domain structure study of the Myddosome [13]. Only 1 to 4 MyD88 molecules bind to TIRAP upon strong LPS stimulation. The stimulus-strength-dependent pattern of MyD88 is consistent with a higher-order assembly study of procaspase-8 in DISC, which implies that procaspase-8 forms a long chain upon weak stimulation, but forms a short chain upon strong stimulation [22,23]. Our study further suggests that the MyD88 higher-order structure is determined by the level of TIRAP in the Myddosome. The downstream signals adopt different aggregation strategies under different circumstances and the possible mechanism is that high-order assembly structure could amplify signaling intensity [15,16] for efficient transduction when the inducer level is low (Detailed information is in Supplementary Materials), which should be generally applicable in different pathways.

Taken together, our study provides an unexpected view on TLR4 complex assembly as well as the higher-order assembly strategy for LPS signaling transduction. Our study is a MS data-based modeling analysis and the main goal is to quantitatively describe the complex assembly processes. This work presents a mechanism elucidation on the strength of complexes and strength dependent stoichiometry and provides important guidance for further experiment analysis. The powerful approach that combines MS-data and mathematical modeling can be further applied to other biology systems for obtaining new insights.

## 4. Materials and Methods

### 4.1. Modeling Principles

All biochemical association/disassociation reactions in LPS/TLR4 complexes assembly processes (Figure 2A) are represented by molecule–molecule interactions:

(1) $$A+B\overset{k^{\mathrm{on}}}{\to }\mathrm{AB}\overset{k^{\mathrm{off}}}{\to }A+B^{*}$$

The interactions are mainly described by a reaction step for the association of complex partners (AB) with the kinetic rate constant kon. The subsequent protein modification is described by the disassociation step with the rate constant koff. Biochemical reaction rates are dependent on protein concentrations and kinetic rate constants depend on the law of mass action. Dynamics of the biochemical reactions and connectivity of signaling molecules can be formulated as a set of coupled ordinary differential equations (ODEs):

(2) $${\mathrm{dx}}_{i}/\mathrm{dt}=\sum_{j=1}^{n}v_{\mathrm{ij}}\cdot q_{j}\;,\;(i=1,\ldots ,m)$$

where m represents the number of components with the concentration xi and dxi/dt is the rate of concentration change with time. n is the number of reactions with the rate qj, and vij denotes the element of stoichiometric matrix that links the reaction rates of qi with component xi. The kinetic parameters (Table 1 and Table 2) are reasonably estimated with biochemical constraints and fixed with a global optimization method that minimizes the deviation between simulation results and SWATH-MS data of the protein’s behaviors. The two models were developed and simulated with MATLAB (Version R2016a) and the corresponding ODEs are respectively presented in Tables S1 and S2.

### 4.2. Parameter Values Determination

There are 17 and 37 total parameters in Model 1 and 2, respectively. The parameters are mostly determined by a global optimization method that minimizes the deviation between simulation results and SWATH-MS data. The deviation is characterized by using the correlation coefficient, R-square, which is determined as the following functions:

(3) $${}^{R^{2}=1-\frac{\sum_{i=1}^{n}{\left(y_{\exp}(t_{i})-y_{\mathrm{sim}}(t_{i})\right)}^{2}}{\sum_{i=1}^{n}{\left(y_{\exp}(t_{i})-\bar{y_{\exp}}\right)}^{2}}}$$

where y_exp_(t_i_) and y_sim_(t_i_) are the MS data and simulated data at time t_i_, respectively. $\bar{y}$_exp_ is the average value of the MS data. As shown in Figure 1D, the MS data of 8 key proteins are selected to optimize the parameters of the two models. For each protein, there are 10 time points. After optimization of parameters, we calculated the R^2^ of the 8 proteins.

## Figures and Tables

**Table 1:** Initial values of the reactions, kinetic parameters and components in Model 1. (A.U. denotes the arbitrary unit).

Number	Reactions	k_i_	Names	Initial Values (A.U.)
1	LPS + TLR4 → LPS_TLR4	1.28×10^−6^ s^−1^	LPS	500
2	LPS_TLR4 → LPS_bind + TLR4_bind	1.0 s^−1^	TLR4	20
3	TLR4_bind + TIRAP → TLR4_bind_TIRAP	9.3×10^−6^ s^−1^	TIRAP	100
4	TLR4_bind_TIRAP → TLR4_bind + TIRAP_bind	1.0 s^−1^	MyD88	1000
5	TIRAP_bind + MyD88 → TIRAP_bind_MyD88	5.6×10^−5^ s^−1^	IRAK1	100
6	TIRAP_bind_MyD88 → TIRAP_bind + MyD88_bind	1.0 s^−1^	IRAK4	100
7	MyD88_bind + IRAK1 → MyD88_bind_IRAK1	4.75×10^−5^ s^−1^	TRAF6	100
8	MyD88_bind_IRAK1 → MyD88_bind + IRAK1_bind	1.0 s^−1^		
9	MyD88_bind + IRAK4 → MyD88_bind_IRAK4	7.98×10^−6^ s^−1^		
10	MyD88_bind_IRAK4 → MyD88_bind + IRAK4_bind	1.0 s^−1^		
11	IRAK1_bind + TRAF6 → IRAK1_bind_TRAF6	7.0×10^−6^ s^−1^		
12	IRAK1_bind_TRAF6 → IRAK1_bind + TRAF6_bind	1.0 s^−1^		
13	TIRAP_bind → TIRAP_drop	5.0×10^−3^ s^−1^		
14	MyD88_bind → MyD88_drop	1.0×10^−2^ s^−1^		
15	IRAK1_bind → IRAK1_drop	2.2×10^−3^ s^−1^		
16	IRAK4_bind → IRAK4_drop	5.62×10^−4^ s^−1^		
17	TRAF6_bind → TRAF6_drop	1.96×10^−3^ s^−1^		

**Table 2:** Initial values of the reactions, kinetic parameters and components in Model 2.

Number	Reactions	k_i_	Names	Initial Values (A.U.)
1	LPS + TLR4 → LPS_TLR4	1.28×10^−6^ s^−1^	LPS	500
2	LPS_TLR4 → LPS_bind + TLR4_bind	1.0 s^−1^	TLR4	20
3	TLR4_bind + TIRAP → TLR4_bind_TIRAP	9.3×10^−6^ s^−1^	TIRAP	100
4	TLR4_bind_TIRAP → TLR4_bind + TIRAP_bind	1.0 s^−1^	MyD88	1000
5	TIRAP_bind + MyD88 → TIRAP_bind_MyD88	6.0×10^−5^ s^−1^	IRAK1	100
6	TIRAP_bind_MyD88 → TIRAP_bind + MyD88_bind	1.0 s^−1^	IRAK4	100
7	MyD88_bind + IRAK1 → MyD88_bind_IRAK1	2.92×10^−5^ s^−1^	TRAF6	100
8	MyD88_bind_IRAK1 → MyD88_bind + IRAK1_bind	1.0 s^−1^		
9	MyD88_bind + IRAK4 → MyD88_bind_IRAK4	1.2×10^−5^ s^−1^		
10	MyD88_bind_IRAK4 → MyD88_bind + IRAK4_bind	1.0 s^−1^		
11	IRAK1_bind + TRAF6 → IRAK1_bind_TRAF6	7.0×10^−6^ s^−1^		
12	IRAK1_bind_TRAF6 → IRAK1_bind + TRAF6_bind	1.0 s^−1^		
13	TIRAP_bind → TIRAP_drop	5.0×10^−3^ s^−1^		
14	MyD88_bind → MyD88_drop	8.0×10^−3^ s^−1^		
15	IRAK1_bind → IRAK1_drop	4.0×10^−3^ s^−1^		
16	IRAK4_bind → IRAK4_drop	3.0×10^−3^ s^−1^		
17	TRAF6_bind → TRAF6_drop	8.0×10^−4^ s^−1^		
18	MyD88_drop + TRAF6_drop → MyD88_drop_TRAF6_drop	1.0×10^−7^ s^−1^		
19	MyD88_drop_TRAF6_drop → MyD88_drop + TRAF6_BIND	1.0 s^−1^		
20	TRAF6_BIND + MyD88_drop → TRAF6_BIND_ MyD88_drop	1.07×10^−5^ s^−1^		
21	TRAF6_BIND_ MyD88_drop → TRAF6_BIND + MyD88_BIND	1.0 s^−1^		
22	TRAF6_BIND + IRAK1_drop → TRAF6_BIND_IRAK1_drop	6.0×10^−5^ s^−1^		
23	TRAF6_BIND_IRAK1_drop → TRAF6_BIND + IRAK1_BIND	1.0 s^−1^		
24	TRAF6_BIND + IRAK4_drop → TRAF6_BIND_IRAK4_drop	2.92×10^−5^ s^−1^		
25	TRAF6_BIND_IRAK4_drop → TRAF6_BIND + IRAK4_BIND	1.0 s^−1^		
26	TRAF6_BIND → TRAF6_DROP	4.0×10^−4^ s^−1^		
27	MyD88_BIND → MyD88_DROP	5.0×10^−4^ s^−1^		
28	IRAK1_BIND → IRAK1_DROP	1.5×10^−3^ s^−1^		
29	IRAK4_BIND → IRAK4_DROP	1.0×10^−3^ s^−1^		
30	TLR4_bind + MyD88 → TLR4_bind_MyD88	1.0×10^−5^ s^−1^		
31	TLR4_bind_MyD88 → TLR4_bind + MyD88_binda	1.0 s^−1^		
32	MyD88_binda + IRAK1 → MyD88_binda_IRAK1	1.8×10^−6^ s^−1^		
33	MyD88_binda_IRAK1 → MyD88_binda + IRAK1_binda	1.0 s^−1^		
34	MyD88_binda + IRAK4 → MyD88_binda_IRAK4	5.0×10^−7^ s^−1^		
35	MyD88_binda_IRAK4 → MyD88_binda + IRAK4_binda	1.0 s^−1^		
36	IRAK1_binda → IRAK1_dropa	1.4×10^−3^ s^−1^		
37	IRAK4_binda → IRAK4_dropa	1.2×10^−3^ s^−1^		

## Supplementary Materials

The following are available online at https://www.mdpi.com/1422-0067/21/9/3061/s1.
