# Supplementary material for: Data-Driven Modeling Identifies TIRAP-Independent MyD88 Activation Complex and Myddosome Assembly Strategy in LPS/TLR4 Signaling
Source: Int J Mol Sci. 2020 Apr 26;21(9):3061. doi: 10.3390/ijms21093061 (PMC7246728; doi:10.3390/ijms21093061)
Supplement: Supplementary file 1 [file ijms-21-03061-s001.pdf]

Generic model higher-order aggregation

Our study show that the MyD88 higher-order structure is determined by TIRAP level in Myddosome, but the question why higher-order aggregation of MyD88 occurs at a low level of TIRAP, but does not apparently occur when TIRAP level is high remains unsolved. To analyse the condition for such changes, we constructed a simple model, where upstream A recruits downstream B, to discuss the effects of A levels and aggregation forms of B on the amount of B recruited by A (Figure S1A).

The reaction of A associates with higher-order aggregation protein B can be described as:

$$A + n \cdot B \xrightarrow{k} AB_n ; \qquad (1)$$

Where n represents the chain length of B in the associated complex. k is the association parameter. Then, ODE that represents the level change of B in complex can be formulated as follows:

$$d[B_{bind}]/dt = n \cdot k \cdot [A] \cdot [B]^n . \qquad (2)$$

Both the initial values of A and B are 10 A.U. Value of the association parameters is 10^{-4} A.U. Denote n to represent the ratio of B to A in complex. Increase of n leads to more B recruited into complex (Figure S1B). Further comprehensive two-dimensional diagram implies that at a low level of A (~ 5-10 A.U.), only a small amount of B is recruited when n=1 (Figure S1C, upper panel). But if B is recruited in the form of dimerization (n=2) or tetramerization (n=4) in complex, B is totally recruited, suggesting a signaling amplification behavior of the higher-order assembly process. However, B can also be totally recruited in complex at a high level of A (> 10 A.U.) when n=1, while there is no obvious difference between the results when n=2 and n=4 (Figure S1C, upper panel) at 60 min. Therefore, these results indicated that, for efficient recruitment and signaling transduction, higher-order aggregation of downstream component is required when the upstream level is low, but it is unnecessary when the upstream level is high enough. Amplification behavior can also be observed under different reaction rates (Figure S1C, down panel). Thus, the higher-order assembly mechanisms should be generally applicable.

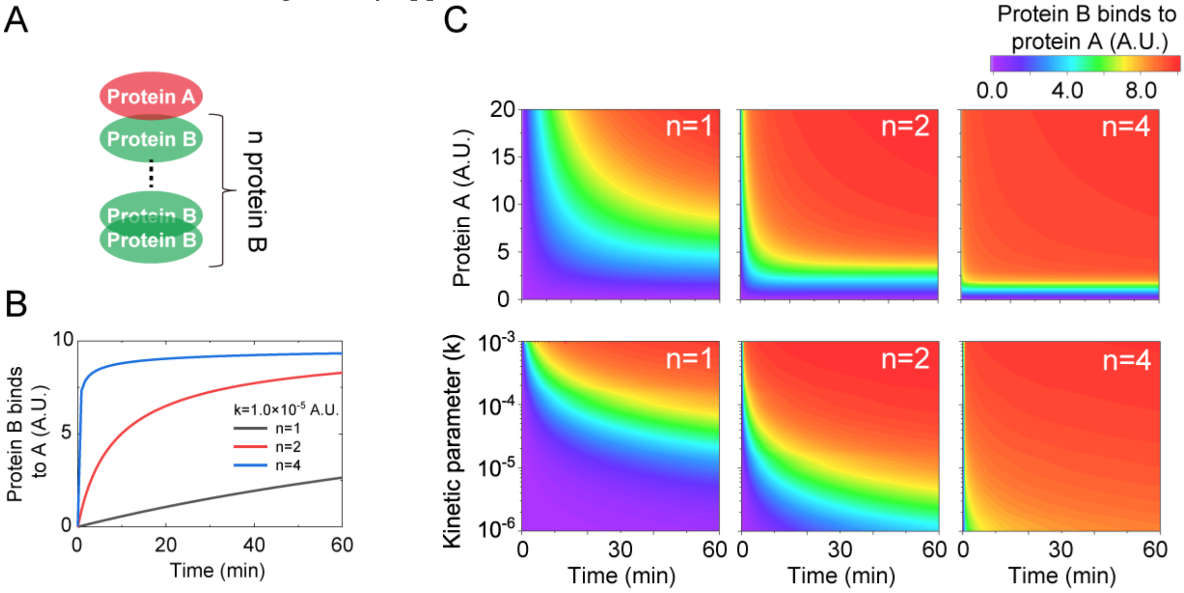

Figure S1. Signaling amplification induced by higher-order assembly. (**A**) Schematic of a simple higher-order assembly model. (**B**) Time course of the amount change of B in complex with different n. (**C**) Two-dimensional diagram indicating the signal amplification function of higher-order assembly with A level changes (upper panel) and association parameter k changes (down panel).

Table S1. Ordinary differential equations (ODEs) in Model 1.

```
d[LPS]/dt=-k1*LPS*TLR4;
d[TLR4]/dt=-k1*LPS*TLR4;
d[LPS_TLR4]/dt=k1*LPS*TLR4-k2*LPS_TLR4;
d[LPS_bind]/dt=k2*LPS_TLR4;
d[TLR4_bind]/dt=k2*LPS_TLR4-k3*TLR4_bind*TIRAP+k4*TLR4_bind_TIRAP;
d[TIRAP]/dt=-k3*TLR4_bind*TIRAP;
d[TLR4_bind_TIRAP]/dt=k3*TLR4_bind*TIRAP-k4*TLR4_bind_TIRAP;
d[TIRAP_bind]/dt=k4*TLR4_bind_TIRAP-k5*TIRAP_bind*MyD88+k6*TIRAP_bind_MyD88-k13*TIRAP_bind;
d[MyD88]/dt=-k5*TIRAP_bind*MyD88;
d[TIRAP_bind_MyD88]/dt=k5*TIRAP_bind*MyD88-k6*TIRAP_bind_MyD88;
d[MyD88_bind]/dt=k6*TIRAP_bind_MyD88-k7*MyD88_bind*IRAK1+k8*MyD88_bind_IRAK1-
   k9*MyD88_bind*IRAK4+k10*MyD88_bind_IRAK4-k14*MyD88_bind;
d[IRAK1]/dt=-k7*MyD88_bind*IRAK1;
d[MyD88_bind_IRAK1]/dt=k7*MyD88_bind*IRAK1-k8*MyD88_bind_IRAK1;
d[IRAK1_bind]/dt=k8*MyD88_bind_IRAK1-k11*IRAK1_bind*TRAF6+k12*IRAK1_bind_TRAF6-k15*IRAK1_bind;
d[IRAK4]/dt=-k9*MyD88_bind*IRAK4;
d[MyD88_bind_IRAK4]/dt=k9*MyD88_bind*IRAK4-k10*MyD88_bind_IRAK4;
d[IRAK4_bind]/dt=k10*MyD88_bind_IRAK4-k16*IRAK4_bind;
d[TRAF6]/dt=-k11*IRAK1_bind*TRAF6;
d[IRAK1_bind_TRAF6]/dt=k11*IRAK1_bind*TRAF6-k12*IRAK1_bind_TRAF6;
d[TRAF6_bind]/dt=k12*IRAK1_bind_TRAF6-k17*TRAF6_bind;
d[TIRAP_drop]/dt=k13*TIRAP_bind;
d[MyD88_drop]/dt=k14*MyD88_bind;
d[IRAK1_drop]/dt=k15*IRAK1_bind;
d[IRAK4_drop]/dt=k16*IRAK4_bind;
d[TRAF6_drop]/dt=k17*TRAF6_bind;
```

Table S2. Ordinary differential equations (ODEs) in Model 1.

```
d[LPS]/dt=-k1*LPS*TLR4;
d[TLR4]/dt=-k1*LPS*TLR4;
d[LPS_TLR4]/dt=k1*LPS*TLR4-k2*LPS_TLR4;
d[LPS_bind]/dt=k2*LPS_TLR4;
d[TLR4_bind]/dt=k2*LPS_TLR4-k3*TLR4_bind*TIRAP+k4*TLR4_bind_TIRAP-
   k30*TLR4_bind*MyD88+k31*TLR4_bind_MyD88;
d[TIRAP]/dt=-k3*TLR4_bind*TIRAP;
d[TLR4_bind_TIRAP]/dt=k3*TLR4_bind*TIRAP-k4*TLR4_bind_TIRAP;
d[TIRAP_bind]/dt=k4*TLR4_bind_TIRAP-k5*TIRAP_bind*MyD88+k6*TIRAP_bind_MyD88-k13*TIRAP_bind;
d[MyD88]/dt=-k5*TIRAP_bind*MyD88-k30*TLR4_bind*MyD88;
d[TIRAP_bind_MyD88]/dt=k5*TIRAP_bind*MyD88-k6*TIRAP_bind_MyD88;
d[MyD88_bind]/dt=k6*TIRAP_bind_MyD88-k7*MyD88_bind*IRAK1+k8*MyD88_bind_IRAK1-
   k9*MyD88_bind*IRAK4+k10*MyD88_bind_IRAK4-k14*MyD88_bind;
d[IRAK1]/dt=-k7*MyD88_bind*IRAK1-k32*IRAK1*MyD88_binda;
d[MyD88_bind_IRAK1]/dt=k7*MyD88_bind*IRAK1-k8*MyD88_bind_IRAK1;
d[IRAK1_bind]/dt=k8*MyD88_bind_IRAK1-k11*IRAK1_bind*TRAF6+k12*IRAK1_bind_TRAF6-k15*IRAK1_bind;
d[IRAK4]/dt=-k9*MyD88_bind*IRAK4-k34*IRAK4*MyD88_binda;
d[MyD88_bind_IRAK4]/dt=k9*MyD88_bind*IRAK4-k10*MyD88_bind_IRAK4;
d[IRAK4_bind]/dt=k10*MyD88_bind_IRAK4-k16*IRAK4_bind;
d[TRAF6]/dt=-k11*IRAK1_bind*TRAF6;
d[IRAK1_bind_TRAF6]/dt=k11*IRAK1_bind*TRAF6-k12*IRAK1_bind_TRAF6;
d[TRAF6_bind]/dt=k12*IRAK1_bind_TRAF6-k17*TRAF6_bind;
d[TIRAP_drop]/dt=k13*TIRAP_bind;
d[MyD88_drop]/dt=k14*MyD88_bind-k18*MyD88_drop*TRAF6_drop+k19*MyD88_drop_TRAF6_drop-
   k20*MyD88_drop*TRAF6_BIND;
d[IRAK1_drop]/dt=k15*IRAK1_bind-k22*IRAK1_drop*TRAF6_BIND;
d[IRAK4_drop]/dt=k16*IRAK4_bind-k24*IRAK4_drop*TRAF6_BIND;
d[TRAF6_drop]/dt=k17*TRAF6_bind-k18*MyD88_drop*TRAF6_drop;
d[MyD88_drop_TRAF6_drop]/dt=k18*MyD88_drop*TRAF6_drop-k19*MyD88_drop_TRAF6_drop;
d[TRAF6_BIND]/dt=k19*MyD88_drop_TRAF6_drop-
   k20*MyD88_drop*TRAF6_BIND+k21*TRAF6_BIND_MyD88_drop-k26*TRAF6_BIND-
   k22*IRAK1_drop*TRAF6_BIND+k23*TRAF6_BIND_IRAK1_drop-
   k24*IRAK4_drop*TRAF6_BIND+k25*TRAF6_BIND_IRAK4_drop;
d[TRAF6_BIND_MyD88_drop]/dt=k20*MyD88_drop*TRAF6_BIND-k21*TRAF6_BIND_MyD88_drop;
d[MyD88_BIND]/dt=k21*TRAF6_BIND_MyD88_drop-k27*MyD88_BIND;
```

d[TLR4_bind_MyD88]/dt=k30*TLR4_bind*MyD88-k31*TLR4_bind_MyD88;
d[MyD88_binda]/dt=k31*TLR4_bind_MyD88-k32*IRAK1*MyD88_binda+k33*MyD88_binda_IRAK1-k34*IRAK4*MyD88_binda+k35*MyD88_binda_IRAK4;
d[MyD88_binda_IRAK1]/dt=k32*IRAK1*MyD88_binda-k33*MyD88_binda_IRAK1;
d[IRAK1_binda]/dt=k33*MyD88_binda_IRAK1-k36*IRAK1_binda;
d[MyD88_binda_IRAK4]/dt=k34*IRAK4*MyD88_binda-k35*MyD88_binda_IRAK4;
d[IRAK4_binda]/dt=k35*MyD88_binda_IRAK4-k37*IRAK4_binda;
d[TRAF6_DROP]/dt=k26*TRAF6_BIND;
d[MyD88_DROP]/dt=k27*MyD88_BIND;
d[IRAK1_DROP]/dt=k28*IRAK1_BIND;
d[IRAK4_DROP]/dt=k29*IRAK4_BIND;
d[IRAK1_dropa]/dt=k36*IRAK1_binda;
d[IRAK4_dropa]/dt=k37*IRAK4_binda;
d[TRAF6_BIND_IRAK1_drop]/dt=k22*IRAK1_drop*TRAF6_BIND-k23*TRAF6_BIND_IRAK1_drop;
d[IRAK1_BIND]/dt=k23*TRAF6_BIND_IRAK1_drop-k28*IRAK1_BIND;
d[TRAF6_BIND_IRAK4_drop]/dt=k24*IRAK4_drop*TRAF6_BIND-k25*TRAF6_BIND_IRAK4_drop;
d[IRAK4_BIND]/dt=k25*TRAF6_BIND_IRAK4_drop-k29*IRAK4_BIND;

Amount of MyD88 in TRAF6 complex:
$MyD88_{TRAF6}$ = MyD88_bind + MyD88_BIND;
Amount TRAF6 of in MyD88 complex:
$TRAF6_{MyD88}$ = TRAF6_bind + TRAF6_BIND;
Amount of IRAK1/4 in TRAF6 complex:
$IRAK1_{TRAF6}$ = IRAK1_bind + IRAK1_BIND;
$IRAK4_{TRAF6}$ = IRAK4_bind + IRAK4_BIND;
Amount of IRAK1/4 in MyD88 complex:
$IRAK1_{MyD88}$ = IRAK1_bind + IRAK1_binda + IRAK1_BIND;
$IRAK4_{MyD88}$ = IRAK4_bind + IRAK4_binda + IRAK4_BIND;
